# Fatal Outcome of COVID-19 Relapse in a Fully Vaccinated Patient with Non-Hodgkin Lymphoma Receiving Maintenance Therapy with the Anti-CD20 Monoclonal Antibody Obinutuzumab: A Case Report

**DOI:** 10.3390/vaccines10071021

**Published:** 2022-06-26

**Authors:** Federica Calò, Lorenzo Onorato, Mariantonietta Pisaturo, Antonio Pinto, Loredana Alessio, Caterina Monari, Carmine Minichini, Manuela Arcamone, Alessandra Di Fraia, Luigi Atripaldi, Claudia Tiberio, Nicola Coppola

**Affiliations:** 1 Department of Mental Health and Public Medicine—Infectious Diseases Unit, University of Campania Luigi Vanvitelli, 80131 Naples, Italy; federica.calo@policliniconapoli.it (F.C.); lorenzo.onorato@unicampania.it (L.O.); mariantonietta.pisaturo@unicampania.it (M.P.); loredana.alessio@policliniconapoli.it (L.A.); caterina.monari@policliniconapoli.it (C.M.); carmine.minichini@alice.it (C.M.); alessandra.difraia@policliniconapoli.it (A.D.F.); 2 Hematology-Oncology and Stem Cell Transplantation Unit, Istituto Nazionale Tumori, Fondazione ‘G. Pascale’, IRCCS, 80131 Naples, Italy; a.pinto@istitutotunori.na.it (A.P.); m.arcamone@istitutotumori.na.it (M.A.); 3 U.O.C. di Patologia Clinica Ospedale D. Cotugno, Azienda Sanitaria Ospedali dei Colli, 80131 Naples, Italy; luigi.atripaldi@ospedalideicolli.it (L.A.); claudia.tiberio@ospedalideicolli.it (C.T.)

**Keywords:** SARS-CoV-2, COVID-19, oncohematological diseases, anti-B cells monoclonal antibodies, mRNA vaccine, relapse

## Abstract

Few data are available regarding the effectiveness of anti-SARS-CoV-2 vaccine in immunocompromised patients. Vaccination may have a suboptimal efficacy in this population, in particular if patients are exposed to anti-B-cell therapy. We report the virological and clinical characteristics of a patient with follicle center lymphoma under bimonthly maintenance therapy with obinutuzumab, an anti-CD20 monoclonal antibody. Despite three doses of BNT162b2 vaccine, the patient was infected by the SARS-CoV-2 Omicron variant. After an initial period of clinical and molecular remission due to early therapy with sotrovimab, the patient experienced a fatal relapse sustained by the same viral strain.

## 1. Background

The ongoing pandemic due to severe acute respiratory syndrome coronavirus 2 (SARS-CoV-2) has rapidly spread worldwide since December 2019, causing approximately 6,252,316 deaths [1].

Common symptoms of SARS-CoV-2 infection include fever, cough, and fatigue, with some cases precipitating to more complex clinical pictures such as acute respiratory distress syndrome with a mortality rate of 10% [2]. The elderly and patients with comorbidities have shown a more severe course of infection [3]. In particular, a worse outcome has been described among oncologic patients [2,4,5], and several studies have demonstrated that male sex, smoking status, and a diagnosis of hematologic malignancies or lung cancer are independently associated with disease severity and mortality in this population [4].

The availability of safe and effective COVID-19 vaccines represents a powerful tool for controlling the pandemic and preventing severe COVID-19, hospitalization, and death. Two doses of BNT162b2 were highly effective in preventing symptomatic SARS-CoV-2 infection [6]. Moreover, recent data have shown how a third dose of the BNT162b2 mRNA vaccine is effective in protecting individuals against severe COVID-19-related outcomes compared with those receiving only two doses [7]. Data on the efficacy of vaccines in a fragile population of patients, such as those suffering from different hematopoietic tumors, is still scarce. Patients with lymphoid cancers have been identified as being particularly at risk of inadequate antibody response to anti-SARS-CoV-2 vaccines [8], particularly those with non-Hodgkin lymphoma (NHL) receiving B cell-depleting agents [9]. Indeed, the impact of different antineoplastic therapies is largely variable; for example, immune check point inhibitors are not associated with a higher mortality risk in COVID-19 patients, unlike CAR T-cell therapies and anti-CD20 monoclonal antibodies [4].

In this context, we report the virological and clinical characteristics of a patient with NHL who, despite three doses of BNT162b2 vaccine, contracted a SARS-CoV-2 infection sustained by the Omicron variant. After an early treatment with sotrovimab yielding to an initial period of remission, the patient experienced a clinical and a virological fatal relapse sustained by the same viral strain.

## 2. Methods

In May 2020, a 53-year-old Caucasian male was diagnosed with a follicle center lymphoma (FCL). Staging procedures highlighted extensive lymphoma dissemination involving supra- and sub-diaphragmatic lymph nodes along with massive paravertebral lymphadenopathies. Imaging procedures disclosed also the involvement of meningeal layers and the cranial theca and of the left upper lung lobe. Due to the high tumor burden and the extranodal presentation with central nervous system involvement, the patient received from June to November 2020 six courses of the anti-CD20 monoclonal antibody obinutuzumab together with CHOP (cyclophosphamide, adryamin, vincristine, prednisone) chemotherapy (G-CHOP), two cycles of methotrexate, and two additional doses of obinutuzumab. At the end of the induction treatment, the patient achieved compete clinical and radiological remission. In January 2021 he started an obinutuzumab bi-monthly maintenance therapy up to December 2021, for a total of six obinutuzumab administrations. During the maintenance phase, the patient underwent anti-SARS-CoV-2 vaccination (mRNA vaccine BNT162b29), with the first, second, and third vaccine administrations given on 15 April, 6 May, and 27 December 2021, respectively.

## 3. Results

**Figure 1:**
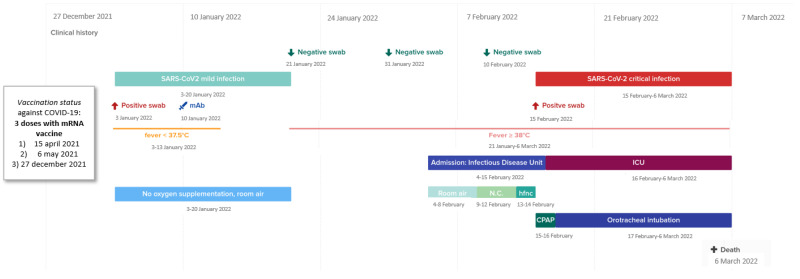
Timeline of the clinical and virological history of thePl SARS-CoV-2 infection. Legend: mAb: monoclonal antibody, NC: nasal cannula, hfnc: high flow nasal cannula, CPAP: continuous positive airway pressure.

On 3 January 2022, a week after the third vaccine dose, the patient developed mild symptoms characterized by low-grade fever and cough for which he underwent a nasal/oro-pharyngeal swab test for SARS-CoV-2, which was positive. The clinical and virological timeline of the COVID-19 course is summarized in Figure 1. In order to prevent the progression of COVID-19, on 10 January 2022 (day +7 from the onset of symptoms), he received an infusion of the anti-SARS-CoV-2 monoclonal antibody sotrovimab, with a rapid remission of symptoms. At the time of the sotrovimab infusion, he tested negative for anti-spike titer (normal value under 33.8 binding arbitrary units/mL; Liaison SARS-CoV-2-trimeric S IgG, Diasorin, Saluggia, Italy). On 21 January, he tested negative for SARS-CoV-2 on the control swab (Figure 1).

However, from the same day of the SARS-CoV-2 negative test, the patient experienced high-grade fever (39 °C) with dyspnea. On January 24 he started antibiotic therapy with ceftriaxone without any clinical benefit. In the same day, a chest X-ray showed infiltrates of parenchymal thickening in the right middle and lower lobe. On January 31, he tested negative for SARS-CoV-2 on the molecular swab once again.

On February 4 he was admitted to the hospital for diagnostic work up of the prolonged febrile illness. At the entrance he appeared hemodynamically stable but tachycardic and feverish with 96% O_2_ saturation on ambient air. The blood test revealed a marked inflammatory state characterized by C-reactive protein (CRP) of 175 mg/L, procalcitonin of 0.60 μg/mL, fibrinogen of 771 mg/dL, ferritin of 544 μg/mL, and D-dimer of 533 μg/mL.

At hospital admission, several diagnostic tests were requested: blood cultures, anti-Human Immunodeficiency Virus, Cytomegalovirus-DNA, Epstein–Barr–DNA, beta-d-glucan, galactomannan, sputum examination for common bacteria and *M. tuberculosis*, Multiplex-PCR respiratory panel for virus and atypical bacterial, and legionella antigen. All these tests were negative. Only quantiferon was found to be positive. A full body CT scan (Supplementary Figure S1) showed multiple bilateral areas of ground glass with crazy paving pattern. The prophylaxis in place with co-trimoxazole and acyclovir was continued, and antibiotic therapy with piperacillin/tazobactam was started and maintained for a total of 7 days.

Starting from 8 February, the patient began to experience dyspnea with oxygen desaturation. On February 10, he tested again negative for SARS-CoV-2 on the molecular test PCR.

On February 13 he was put on a high flow nasal cannula (HFNC), 50% FiO_2_, 60 L/min, due to severe respiratory failure (Pa02/FiO2 ratio of 163 from the arterial blood gas analysis).

Due to the worsening respiratory symptoms, a chest CT scan and fibrobronchoscopy were obtained. The CT scan showed large areas of bilateral and symmetrical ground glass with crazy paving pattern, without, however, opacification defects of the pulmonary arteries (Supplementary Figure S1). Furthermore, considering the clinical and radiological picture, a further swab for the search for SARS-CoV-2 was analyzed and at this time was positive with the threshold cycle (CT) of the RT-PCR of 27 for ORF 1ab and 28 for N and for E genes. That same day, SARS-CoV-2-antigen was detected in the serum at high load (178 pg/mL) by chemiluminescent enzyme immunoassay (Lumipulse-G SARS-CoV-2-Ag, Fujirebio Holdings, Tokyo, Japan). Moreover, the specimen collected during the fibrobronchoscopy was negative for tuberculosis and common bacterial culture, but positive for SARS-CoV-2-RNA.

Despite the HFNC, the patient presented desaturations and was therefore placed in a helmet with continuous positive airway pressure (CPAP), 40% FiO_2_, and positive end expiratory pressure (PEEP) equal to 8 cm H_2_O. Moreover, a blood test showed total immunoglobulins IgG and IgM of 194 mg/dL (range 800–1800) and <19.2 mg/dL (range 80–280), respectively, and persistence of an inflammatory state characterized by CRP 170 mg/L, procalcitonin 0.30 μg/mL, fibrinogen 542 mg/dL, and D-dimer 5016 μg/mL.

Due to progressive respiratory failure (Pa02/FiO2 ratio of 85 at arterial blood gas analysis), on February 16 he was transferred to the intensive care unit (ICU), where he underwent orotracheal intubation and received, while pending the results of the microbiological tests performed during the fibrobronchoscopy, broad-spectrum antibiotics, anti-*Pneumocystis jirovecii* and herpes-virus prophylaxis, corticosteroid, and tocilizumab treatment. On March 4, the COVID-19 surveillance swab was still positive. After a few days of ICU stay, he developed several infectious complications, and, subsequently, a septic shock by mechanical ventilation-associated pneumonia, which led to death on 6 March.

**Figure 2:**
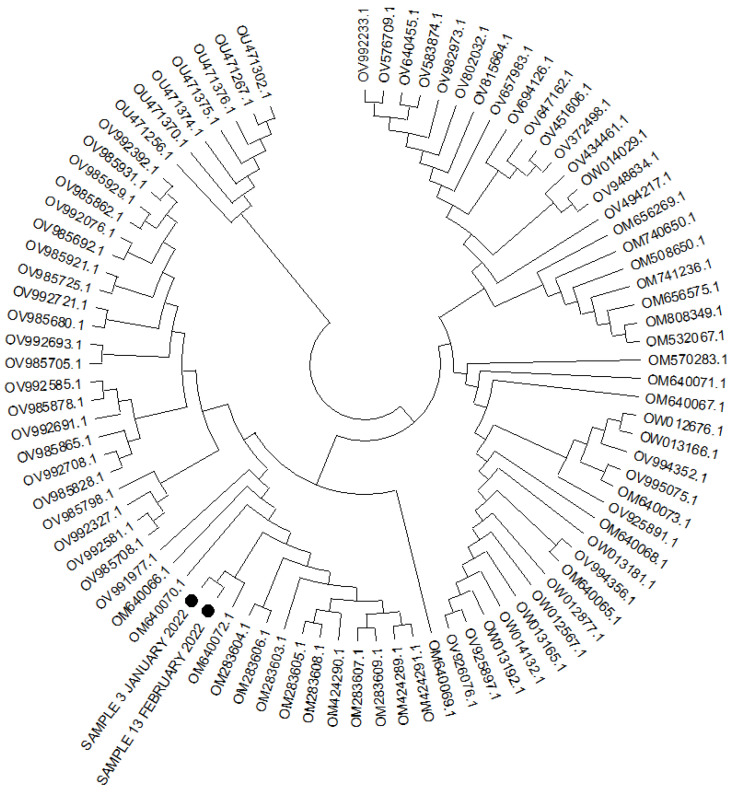
Phylogenetic tree for 89 complete genome of the SARS-CoV-2 (lineage B.1.1.529-BA.2) using the neighbor joining and 1000 bootstrap method. Legend: the black circles indicate two cases analyzed.

The analysis of the SARS-CoV-2 RNA (Figure 2) isolated on 10 January 2022 (first course of SARS-CoV-2 infection at the day of sotrovimab infusion) and on 15 February 2022 showed the presence of the same viral clade, the VOC-21NOV-01 (B.1.1.529) variant, named the Omicron variant. In addition, neighbor joining, and maximum likelihood methods were carried out for similarity assessment and evolution analysis (Figure 2).

## 4. Discussion

We reported the case of an FCL patient extensively treated with the anti-CD20 monoclonal antibody obinutuzumab who showed recurring SARS-CoV-2-related pneumonia that led to fatal respiratory failure. The patient experienced a critical illness leading to *exitus* despite the receipt of a full three-dose schedule of anti-SARS-CoV-2 mRNA vaccine and a therapy with sotrovimab at day 7 after the first onset of symptoms.

Several studies have reported that patients with solid or hematological malignancies present a more severe clinical episode of COVID-19 disease compared to non-cancer patients [10]. In particular, patients with hematological neoplasms present a significantly higher mortality rate as compared to subjects affected by solid tumors [10].

In addition, patients with lymphoproliferative diseases can present prolonged viral shedding and persistent infection [11,12], especially those receiving antineoplastic chemotherapy during the previous 30 days, and treatment with anti-CD20 antibodies or cellular therapy during the last year [12]. A recently published study reported the cases of four patients with lymphoproliferative diseases, all treated with anti-CD20 antibodies, showing recurrent COVID-19 pneumonia and prolonged PCR positivity [11]. No data are available regarding the vaccination status of the patients; however, none of them tested positive for SARS-CoV-2 serology at hospital discharge.

Thus, in the pre-vaccination era, ongoing treatment with anti-CD20 antibodies was significantly associated with poor outcomes upon COVID-19 development and impaired anti-viral endogenous humoral response [13].

Although COVID-19 vaccination has been demonstrated to significantly reduce the infection rate and the related deaths in patients with cancer, limited effectiveness has been described in the subgroup of subjects affected by hematologic malignancies. The CANVAX study showed that immune responses to SARS-CoV-2 vaccines are modestly impaired in patients with cancer, suggesting the utility of antibody testing to identify patients for whom additional vaccine doses may be effective and appropriate [14]. A suboptimal humoral response to two doses of BNT162b2 or AZD1222 vaccines has been reported in a cohort of 585 cancer patients, with a seroconversion rate of 59% after the second dose in patients with hematological neoplasms as compared to 85% in subjects with solid tumors [15]; furthermore, patients with hematological malignancies had lower median neutralizing antibodies than those with solid cancers against both SARS-CoV-2 wild-type and Alpha, Beta, and Delta variants.

Limited data are available on the efficacy of a three-dose schedule against the Omicron variant in this population. A study conducted among 50 patients with solid tumors reported a significantly higher neutralizing capacity in those who received a booster dose compared to the recipients of only two doses of mRNA vaccine [16]. However, it is unclear whether these findings can be extended to patients with hematological neoplasms.

Our patient started and completed the sequence of three BNT162b2 vaccine doses during bi-monthly maintenance anti-CD20 administration. The first vaccine dose was delivered after 10 previous infusions of obinutuzumab and the last after a total of 16. It was previously shown that anti-CD20 administration severely impairs humoral response to COVID-19 vaccines in NHL patients; indeed, 50% of these patients are unable to develop significant levels of SARS-CoV-2 blocking antibodies as compared with healthy controls [8]. More importantly, time since last anti-CD20 administration emerged as a significant independent predictor of humoral response to vaccination. In our patient, both the high load of anti-CD20 treatments and the lack of any substantial time lag between obinutuzumab and vaccination might have concurred to render three administrations of BNT162b2 unable to elicit clinically significant levels of blocking of anti-viral antibodies, leading to the fatal outcome of his SARS-CoV-2 infection. As a matter of fact, he was negative for anti-spike titer at the time of first symptoms development, showing the inability of its immune system to produce an effective humoral response against the virus. For further development of the research, it would be interesting to investigate the antibody titer of vaccinated people receiving anti-CD20 therapy in comparison to healthy vaccinated people in order to compare the correlation between antibody responses and fatal outcomes.

A further point to consider is that the patient experienced a resolution of symptoms and viral clearance of nasopharyngeal swab after the administration of sotrovimab. A prospective multicenter study demonstrated that bamlanivimab and casirivimab/imdevimab were effective in reducing disease progression and mortality among hematological patients in early stages of SARS-CoV-2 infection [17]. However, data on the efficacy of sotrovimab in cancer patients infected with the Omicron variant, especially after sustained exposure to therapeutic anti-CD20 antibodies, are still lacking. Lastly, another interesting topic of our case is that despite the molecular nasopharyngeal swab being negative shortly after treatment with sotrovimab, peripheral blood antigenemia was persistently positive.

An additional treatment option for immunocompromised patients with COVID-19 is represented by convalescent plasma therapy, which has been demonstrated to significantly reduce the 30-day mortality at the propensity score-adjusted analysis in a large cohort of patients with hematologic cancer [18]. A recent review of the literature has reported encouraging clinical outcomes in COVID-19 patients with severe depletion of B-cell lymphocytes because of anti-CD20 therapy that were treated with convalescent plasma [19]. However, most of the data derived from case series. Larger prospective studies are needed to evaluate the role of convalescent plasma in this particular setting.

## 5. Conclusions

SARS-CoV-2 infection can cause prolonged and severe diseases in cancer patients, and vaccinations show suboptimal efficacy in this immunocompromised population. The best strategies for the prevention and treatment of COVID-19 in this setting are still a matter of debate. Every effort should be made to vaccinate the majority of patients before treatment initiation. In fact, patients who started anti-CD20 soon after having achieved a vaccine-induced anti-viral humoral response were shown to usually maintain clinically significant blocking antibody levels throughout subsequent anti-CD20 administrations.

## Data Availability

The data presented in this study are available on request from the corresponding author.

## Supplementary Materials

The following are available online at https://www.mdpi.com/article/10.3390/vaccines10071021/s1, Figure S1: Radiological characteristics and comparison between the CT scan of 5 and 14 February 2022.
