# Supplementary material for: Fatal Outcome of COVID-19 Relapse in a Fully Vaccinated Patient with Non-Hodgkin Lymphoma Receiving Maintenance Therapy with the Anti-CD20 Monoclonal Antibody Obinutuzumab: A Case Report
Source: Vaccines (Basel). 2022 Jun 26;10(7):1021. doi: 10.3390/vaccines10071021 (PMC9323164; doi:10.3390/vaccines10071021)

Fatal Outcome of COVID-19 Relapse in a Fully Vaccinated Patient with Non-hodgkin Lymphoma Receiving Maintenance Therapy with the ANTI-CD20 Monoclonal Antibody Obinutuzumab: A Case Report

Supplementary Data

Molecular methods

The RNA was extracted from the nasopharyngeal swabs of the patient using QIAamp Viral RNA Mini Kit (Qiagen, Hilden, Germany) following manufacturer's protocol. Both the concentration and the quality of all isolated RNA samples were measured and checked with the Nanodrop2000 (Thermo Fisher Scientific, Waltham, United States)

Viral genomes were amplified by using a multiplex approach, using version 1 of the CleanPlex SARS-CoV-2 Research and Surveillance Panel (Paragon Genomics, Hayward, United States), according to the manufacturer's protocol starting with 50ng of total RNA and followed by Illumina sequencing on a NextSeq 500 (Illumina, san Diego, United States).

Libraries were checked using High Sensitivity Labchip and quantified with Qubit Fluorometric Quantitation system (Thermo Fisher Scientific, Waltham, United States). Raw data were trimmed and analyzed using popular bioinformatics software CLC workbench 5, and Basic Local Alignment Search Tool (BLAST). Italian sequences submitted into GeneBank database (https://www.ncbi. nlm.nih.gov/genbank) from January 2022 to March 2022 and released accession numbers were used to draw phylogenetic trees. Moreover, MEGA X software was used for multiple sequence alignment (MSA) and the phylogenetic trees were drawn using the 1000 replicate bootstrap method.

Supplementary Figure S1: Radiological characteristics and comparison between the CT scan of 5 and 14 February 2022

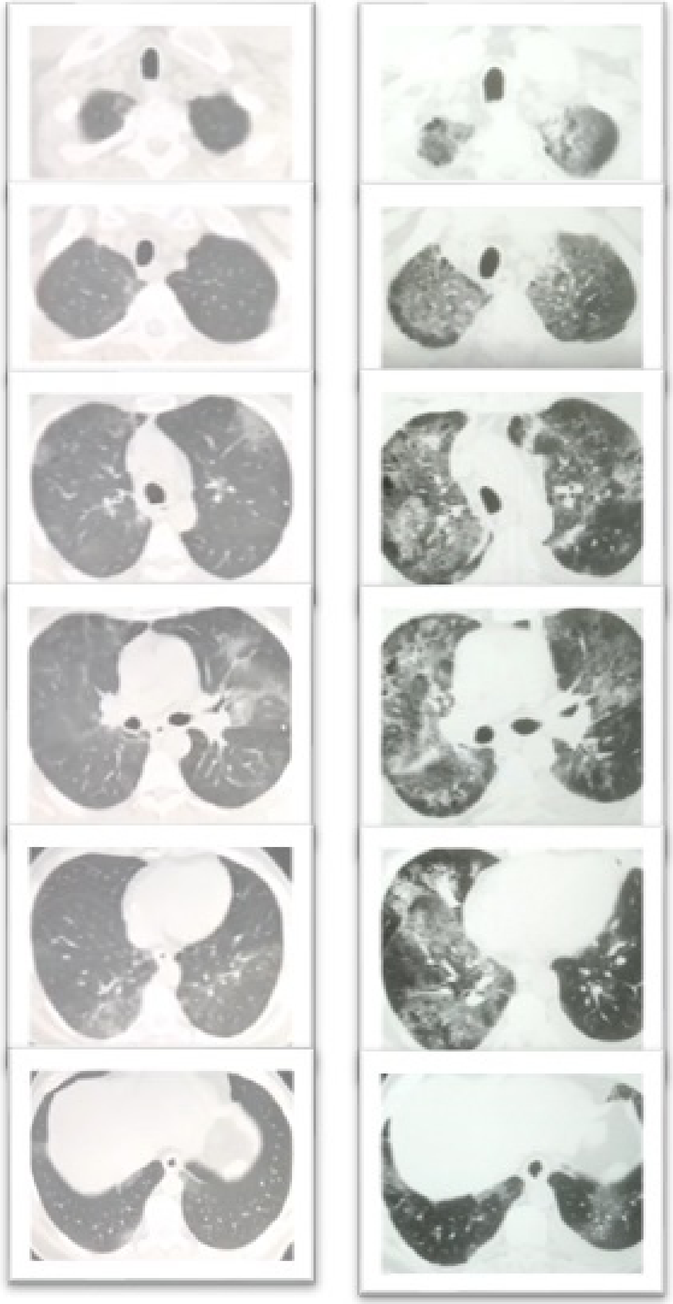

Supplement: Supplementary file 1 [file vaccines-10-01021-s001.zip › vaccines-1752956-supplementary.pdf]
